# Nanostructured Superhydrophobic Titanium-Based Materials: A Novel Preparation Pathway to Attain Superhydrophobicity on TC4 Alloy

**DOI:** 10.3390/nano12122086

**Published:** 2022-06-17

**Authors:** Yuxin Wang, Jiahuan Chen, Yifan Yang, Zihan Liu, Hao Wang, Zhen He

**Affiliations:** 1 School of Materials Science and Engineering, Jiangsu University of Science and Technology, Zhenjiang 212100, China; ywan943@163.com (Y.W.); 15606107161@163.com (J.C.); yyf1875411163000@163.com (Y.Y.); tumingtech@126.com (Z.L.); joyce.haohao@163.com (H.W.); 2 Foshan (Southern China) Institute for New Materials, Foshan 528247, China

**Keywords:** titanium alloy, nanostructure, superhydrophobicity, anodic oxidation

## Abstract

This study develops the nanostructured superhydrophobic titanium-based materials using a combined preparation method of laser marking step and the subsequent anodizing step. The structural properties were determined using an X-ray diffractometer (XRD) and scanning electron microscope (SEM), while the performance was explored by wear and corrosion tests. The laser marking caused a rough surface with paralleled grooves and protrusions, revealing surface superhydrophobicity after organic modification. The anodizing process further created a titanium oxide (TiO_2_) nanotube film. Both phase constituent characterization and surface elemental analysis prove the uniform nanofilm. The inert nanosized oxide film offers improved stability and superhydrophobicity. Compared to those samples only with the laser marking process, the TiO_2_ nanotube film enhances the corrosion resistance and mechanical stability of surface superhydrophobicity. The proposed preparation pathway serves as a novel surface engineering technique to attain a nanostructured superhydrophobic surface with other desirable performance on titanium alloys, contributing to their scale-up applications in diverse fields.

## 1. Introduction

Superhydrophobic surfaces extensively exist in nature, including lotus leaf, butterfly wing, and sharkskin [1,2]. Superhydrophobicity generally refers to a water-repellent surface with a water contact angle (WCA) greater than 150°. Such surface features correlate to some unique properties such as self-cleaning [3,4], oil-water separation [5,6], and anti-icing and anti-fog properties [7,8]. Surface roughness and surface free energy are two vital factors determining surface superhydrophobicity [9]. The construction of superhydrophobic surfaces on alloy surfaces generally requires two steps: generating a rough surface structure, and the following chemical modification to reduce the surface energy.

So far, many previous reports have investigated the superhydrophobic surfaces on metals and their alloys [10]. For instance, Liu et al. [11] prepared a porous microstructure on AZ31 magnesium alloy using the micro-arc oxidation method, and then modified the samples using a stearic acid ethanol solution to attain superhydrophobicity. Yin et al. [12] combined electrochemical deposition and hydrothermal treatment to construct a nanorod-like superhydrophobic coating on stainless steel. The prepared coating possessed good self-cleaning properties and could degrade organic molecules under ultraviolet radiation, thereby preventing the damage from organic pollutants in practical conditions [12].

Titanium alloy shows high specific strength, good heat resistance, great mechanical stability, and corrosion resistance [13,14,15]. In general, constructing a superhydrophobic surface on the light alloys augments the coating corrosion resistance and, more importantly, provides unique functions that expand their application fields. There are some developed methods to prepare superhydrophobic surfaces on titanium alloys, such as electrochemical deposition [16,17], spraying [18], hydrothermal processing [19,20], anodic oxidation [21], and micro-arc oxidation [22]. For example, He et al. [17] deposited a dendritic Zn/ZnO/TiO_2_ film on titanium alloy by electrochemical deposition, whereas the surface superhydrophobicity can be transformed into superhydrophilicity by ultraviolet irradiation. In another report, Sun et al. [23] used dual chemical treatments to prepare superhydrophobic surfaces on different light alloy materials. However, most superhydrophobic surfaces prepared on titanium alloy suffer from their weak mechanical performance, which gradually degrades the superhydrophobicity in a long-term service life [24].

Laser treatment is a facile, fast, and ecologically friendly technology [25]. Laser marking prepares the superhydrophobic surface in recent reports of titanium alloy modification [26,27]. Pu et al. [28] used laser marking to build a superhydrophobic surface on the Ti-6Al-4V (TC4) titanium alloy surface, showing a good abrasion resistance during friction tests. Laser marking is easy to operate, fabricating the controllable micro/nano structure. However, the laser marking process could damage the coating uniformity and decrease surface features’ stability. In another report, micro/nano-structures transition on TC4 alloy surface was studied by sandblasting and anodic oxidation. The authors reported hydrophobic surface of the compact TiO_2_ film, yet more tailoring work is necessary to improve the corrosion resistance and hydrophobicity of the nanotubular surface [29]. Therefore, the study of constructing a stable and superhydrophobic surface in titanium alloys is of great importance. The titanium products with desirable superhydrophobic properties promote their applications in multiple fields, especially in aerospace engineering where the aircraft and spacecraft search for lightweight alloy materials with favorable hydrophobic performance [30,31].

This study develops the nanostructured superhydrophobic titanium-based materials using a preparation method combining laser marking and anodizing. The anodizing process produces a thin, uniform nanotube film on the laser-marked surface, thereby delivering enhanced superhydrophobicity, corrosion resistance, and abrasion resistance. We propose an easy and low-cost method which allows for hydrophobic modification of the surface of titanium alloy products.

## 2. Materials and Methods

### 2.1. Preparation Procedures

The analytical grade chemicals were bought from Aladdin Reagent, China. TC4 (Ti-6Al-4V) titanium alloy sheet with a size of 20 mm × 20 mm × 1 mm was selected as the substrate specimen. The specimens were first polished with 400#, 800#, 1200#, 1500#, 2000# sandpaper to obtain a smooth surface without oxide. Then, ultrasonic cleaning was performed in acetone, absolute ethanol, and deionized water separately for 10 min to remove the debris and grease.

**Figure 1:**
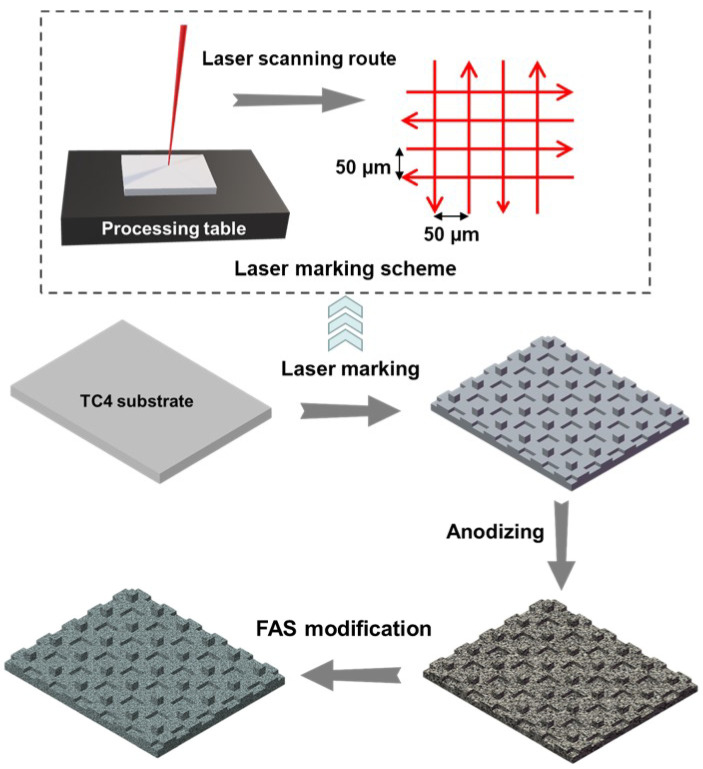
Preparation pathway (i.e., laser marking, anodizing, and FAS modification) for the superhydrophobic surface on TC4 alloy substrate.

The whole sample preparation scheme is illustrated in Figure 1. The pre-treated sample was placed on the processing table and then processed using a fiber laser marking machine (Byes-30W, Bangyi Precision Measuring Instrument Co., Ltd., Shanghai, China) with a wavelength of 1064 nm. The high energy impact produced by the laser beam will melt the titanium alloy that are then cooled and solidified into surface oxides. The surface microstructure can be tailored by adjusting the laser process parameters such as scanning speed, power, and routes. In our study, the laser scan follows grid routes with the same horizontal and vertical scanning interval distance of 50 µm, as shown in Figure 1. The laser power used was 5% with a frequency of 20 kHz, and the scanning rate is 100 mm/s. The specimens were carefully cleaned after constructing a grid-like structure on the surface of the TC4 alloy.

The anodizing process was carried out using a 25 V DC power supply at room temperature. The laser marked TC4 specimen was the anode, and the graphite plate was the cathode. The samples were immersed in a mixed solution of 30 mL/L H_3_PO_4_, 6 g/L H_2_C_2_O_4_ and 6 g/L NaF. The anodic oxidation time was set as 40 min. Finally, the FAS (Fluorosilane, 1H, 1H, 2H, 2H-Perfluorodecyltrimethoxysilane) modification was performed on the as-prepared samples. These samples were immersed in a 1 wt.% FAS ethanol solution for two hours, and then heated in the muffle furnace at 150 °C for 20 min. In order to study the effect of anodizing step, the comparison samples were prepared following the similar preparation scheme in Figure 1 without the anodizing step as well.

### 2.2. Sample Characterization

The scanning electron microscope (SEM, ProX, Phenom, Eindhoven, The Netherlands) was used to observe the sample’s microstructure. The 3D morphology and roughness of the sample surface were explored by laser confocal (VR-6000, Keyence Co., Ltd., Shanghai, China). The X-ray diffractometer (XRD-6000X, Shimazu, Kyoto, Japan), at a step size of 0.1°/s, was used to analyze the phases of the samples before and after anodization. The X-ray photoelectron spectroscopy (XPS, ESCALAB 250Xi, Thermo Fisher, Waltham, MA, USA) was used to analyze the sample surface before and after the FAS modification. The Al Kα radiation at 1486.6 eV was used in the measurements.

The contact angle measuring instrument (SDC-350, Dongguan Shengding Precision Instrument Co., Ltd., Dongguan, China) was used to determine the surface water contact angle (WCA) by dropping 5 μL of water droplets on the surface. The electrochemical workstation (CS2350H, Wuhan Corrtest Instruments Co., Ltd., Wuhan, China) was used to compare and analyze the corrosion resistance of the samples at different processing stages. The abrasion resistance of the superhydrophobic surface was measured by rubbing the sample with sandpaper. The test solution was 3.5% NaCl solution, and the polarization curve ranged from −1 V to 1.5 V.

## 3. Results and Discussion

### 3.1. Microstructure and Phase Constituent

**Figure 2:**
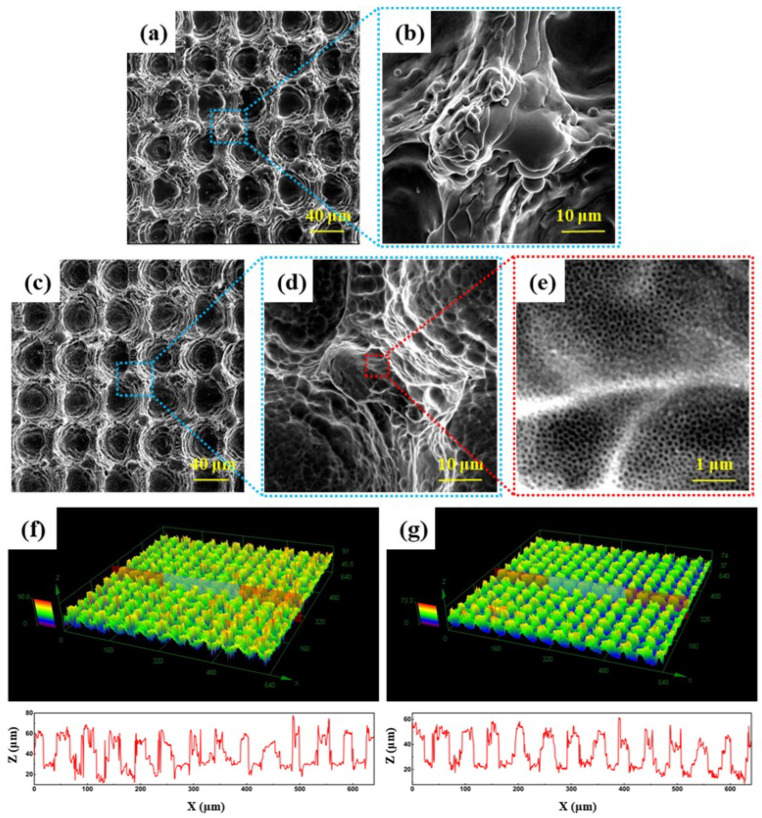
SEM image and 3D topography of samples after laser marking (**a**,**b**,**f**), and samples after laser marking & anodizing (**c**–**e**,**g**), the zoom-in morphologies are indicated by dash-lined squares in the right-side.

Figure 2 presents the surface microstructures observed on the laser-marked sample. After the laser treatment on TC4 alloys, the uniform surface structure consisting of grooves and protrusions is generated as depicted in Figure 2a,b. During the laser marking process, the titanium alloy undergoes the melting/solidification process due to laser heating. The melted materials on the laser scanning path are solidified and piled up on both sides of the laser scanning route. Such rough morphology results from the grid-like laser routes during laser marking processing.

The anodizing treatment constructs nano-structural features on the laser-marked surface. The anodizing treatment barely changes the microstructures of the laser-marked sample (Figure 2c), whereas numerous nano-sized pores are generated on the original surface as shown in Figure 2d,e. The porous nanotube film is fabricated during the anodizing processing on the rough laser-marked surface [32]. The balance between the anodic oxidation and chemical etching grows the TiO_2_ nanotube and generates the nano-pitted morphologies on the sample surface. Figure 2f,g further reveals the 3D surface morphology and roughness recorded on these samples with or without anodizing step. Similar 3D surface images are observed on all samples. The distance between the surface peaks is ~50 µm, which is the same as the interval instance of the laser scanning route. The anodized sample shows a surface peak height at 40–60 µm, which is lower than the laser-marked sample’s peak height at 40–70 µm. In the anodizing step, the chemical etching process consumes a certain extent of surface materials, giving rise to these relatively smooth surface peaks on the anodized samples.

**Figure 3:**
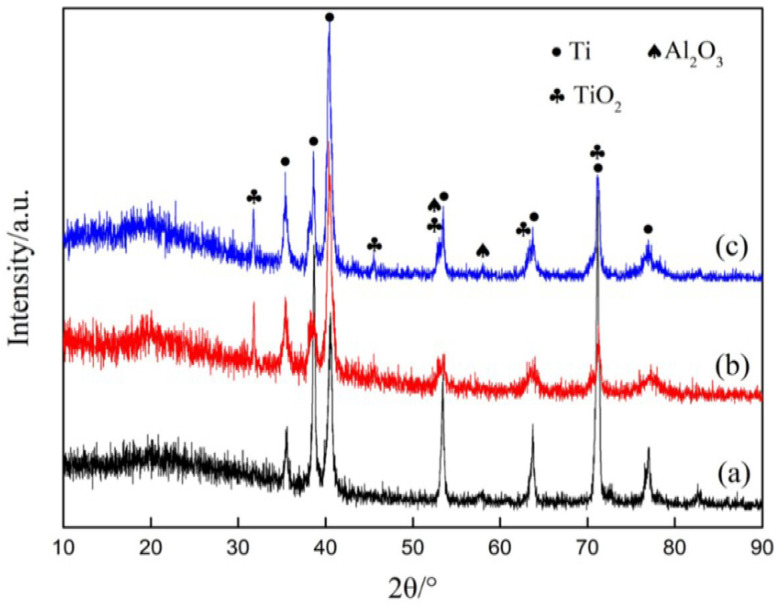
XRD patterns of (**a**) the polished TC4 alloy substrate, (**b**) the sample after laser marking, and (**c**) the sample after laser marking and anodizing (Al_2_O_3_ and TiO_2_ peaks are overlapped in several intense peaks).

To examine the phase variation in the laser marking and anodizing treatments, the phase constituents detected by XRD tests are shown in Figure 3. All these samples display the same diffraction peaks at 35.09°, 38.42°, 40.17°, 53.004°, 62.94°, 70.66°, 76.21°, corresponding to the (100), (002), (101), (102), (110), (103), (112) crystal planes of Ti (PDF#44-1294) that originates from the TC4 alloy substrates. Compared to the untreated substrate, the XRD profile of the treated samples shows several peaks appearing at 31.46°, 45.40°, 52.29°, 62.72°, 71.21°, corresponding to the (111), (112), (220), (113), (041) planes of TiO_2_ (PDF#21-1236), whereas the additional anodizing process barely changes the XRD profiles. This indicates that the surface oxidation reactions occur in both laser marking and anodizing treatments. In addition to TiO_2_, a small content of Al_2_O_3_ is produced at the same time due to the oxidation of aluminum in the TC4 alloy. We should note the following FAS modification step causes no change on the phase constituents of all samples.

**Figure 4:**
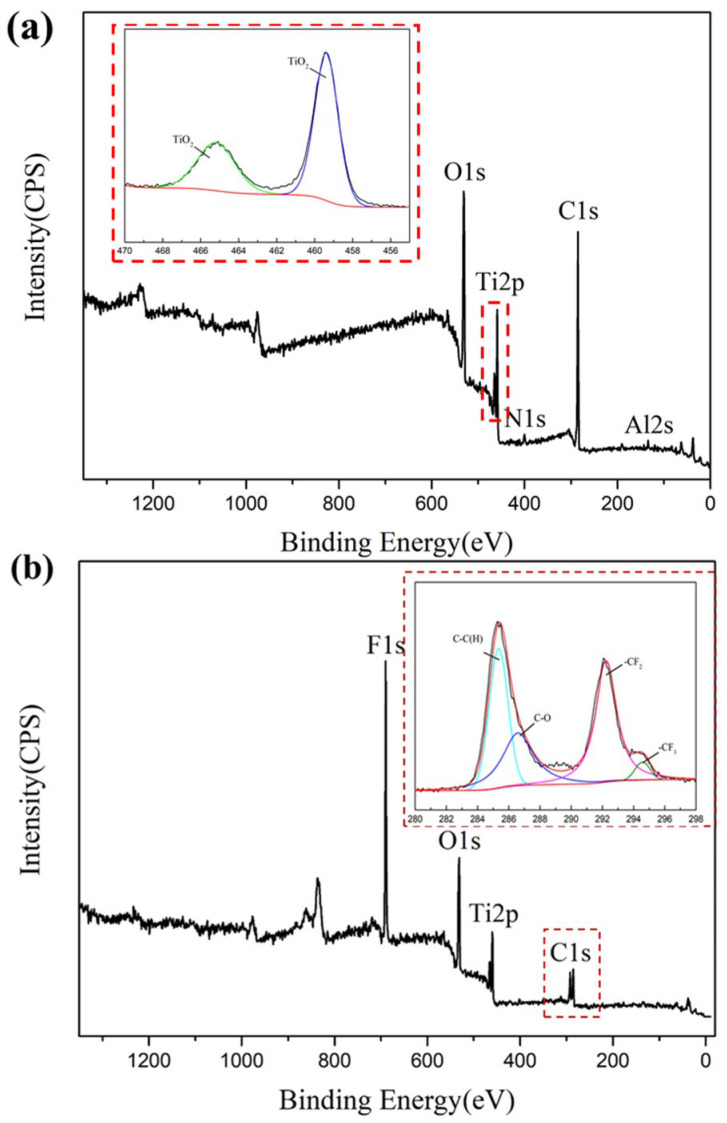
XPS spectra of the sample after the laser marking and anodizing (**a**) before FAS modification, and (**b**) after FAS modification; the insets are the enlarged image of chosen areas.

X-ray photoelectron spectroscopy (XPS) was used to determine the sample surface before and after FAS modification, as depicted in Figure 4. The enlarged Ti spectrum embedded in Figure 4a proves the generation of TiO_2_ in the prepared samples. It can be clearly seen that the additional F1s peaks appear on the sample after FAS modification, Figure 4b. Additionally, the functional groups such as -CF_3_ and -CF_2_ appear in the C1s high-resolution spectrum in Figure 4b, which indicates that the low free energy molecular chains containing fluorine have been attached to the sample surface. The surface superhydrophobicity depends on the low surface free energy. The fluorine-containing molecules is composed of polar end-groups and a long hydrophobic chain. After the FAS modification, the self-assembled low-energy groups of FAS on the surfaces attain surface superhydrophobicity for these samples [33].

### 3.2. Wettability and Corrosion Behavior

**Figure 5:**
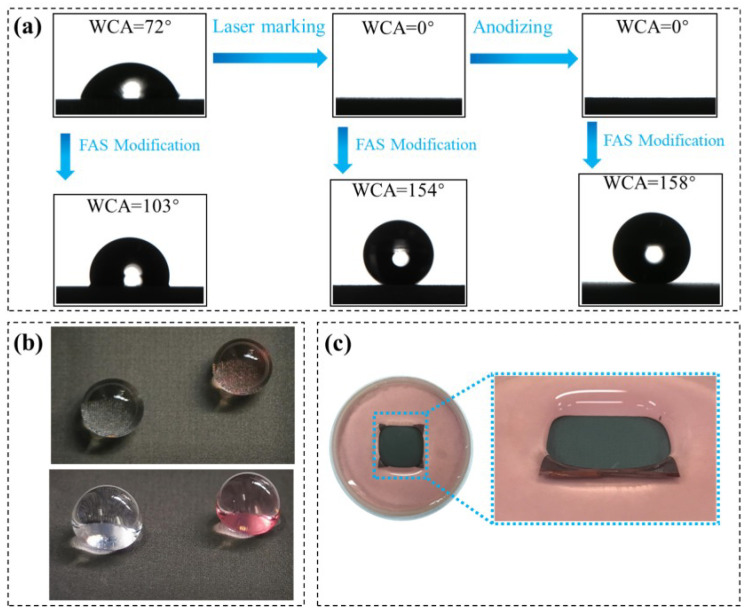
Wetting behavior of the prepared samples: (**a**) surface wettability of samples at different stages, (**b**) the water droplets on the modified surface after laser marking and anodizing and (**c**) the sample immersed in water after laser marking and anodizing, showing a water-free surface below the water level.

The water contact angle (WCA) is detected for these sample surfaces to characterize the wettability, as presented in Figure 5. Generally, the surface is hydrophobic when WCA is larger than 90°, whereas a superhydrophobic surface corresponds to a WCA value greater than 150°. The WCA on the samples at different processing stages is compared in Figure 5a. The WCA on the untreated TC4 alloy surface is 72°, corresponding to a hydrophilic surface. After laser marking treatment, the surface WCA decreases to 0°, revealing a superhydrophilic surface. Additionally, the WCA of the sample after the further anodizing step remains to be 0°. The Wenzel theory explains the above phenomena: the hydrophilicity of the hydrophilic surface increases along with the increases in surface roughness [34]. The laser marking treatment constructs a rough microstructure on the TC4 alloy surface, greatly increasing the surface roughness and reducing the WCA value. The use of FAS modification reduces the surface free energy and provides superhydrophobicity. After FAS modification, the WCA is 103° for the untreated sample, transforming the original hydrophilic surface into a hydrophobic surface. The sample after laser marking attains the superhydrophobic surfaces with the WCA of 154°, and the further anodizing step increases the WCA to be 158°. We should note the fabricated titanium-based surface reveals a higher WCA than the laser-treated sample and anodized sample in earlier reports, indicating its improved superhydrophobicity awarded from the combined process [28,29].

Figure 5b shows the state of a water droplet on the modified surface after laser marking and anodizing. The water droplet remains spherical since the sample surface is water-repellent. At the same time, TC4 alloy substrate becomes grey after laser marking and anodizing, due to the surface oxidation reactions. The superhydrophobic sample is also immersed in water, as depicted in Figure 5c. Even though the sample surface is lower than the water surface, water cannot diffuse over the superhydrophobic surface. This further proves the strong water repellency of the prepared superhydrophobic surface.

The WCA value largely depends on the wetting state on the surface. The most accepted Cassie theory describes the surface status using the equation below [34]:

cosθ_c_ = f_1_ (cosθ_1_ + 1) − 1 (1)

where θ_c_ is the apparent contact angle of the droplet, θ_1_ is the intrinsic contact angle of the liquid on the solid surface, and f_1_ is the ratio of the liquid-solid contact area to the total contact area. In this case, θ_1_ is a constant value since the intrinsic contact angle refers to the contact angle of the liquid drop on an ideal smooth solid surface. After laser marking, the surface generates microstructures where numerous protrusions could support the droplets and prevent the droplets from entering the surface grooves. This produces a large number of air pockets between the droplets and the grooves on the surface. Therefore, the value of f_1_ decreases and the contact angle θ_c_ increases significantly to be 154°. When the anodizing treatment further processes the laser-marked surface, the rough microstructure on the surface will be covered with a thin layer of TiO_2_ nanotubes. This increases the number of air pockets in each nanotube structure and further reduces the value of f_1_. Therefore, the additional anodizing treatment after laser marking can further improve the surface superhydrophobicity.

**Figure 6:**
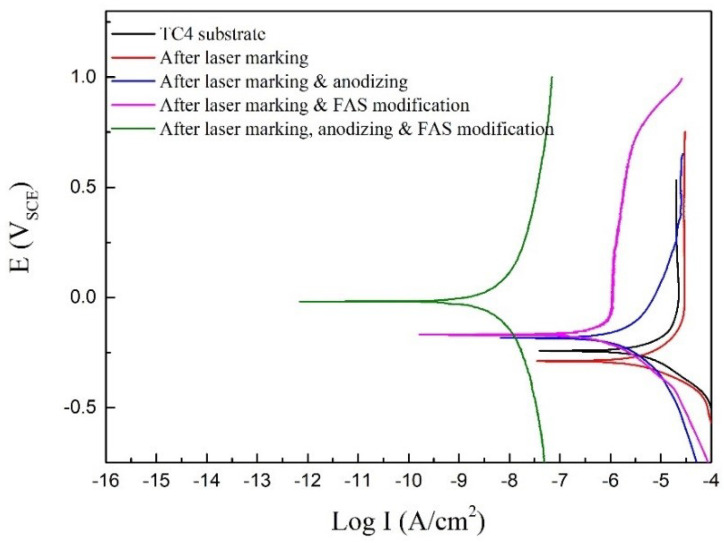
Tafel polarization curves recorded on the samples at different processing stages, tested in 3.5 wt% NaCl solution.

The corrosion behavior poses great significance in industrial applications [35,36]. Figure 6 shows the Tafel polarization curves recorded on the samples at different processing stages, and the calculated parameters are listed accordingly in Table 1. The corrosion potential (E_corr_) of TC4 substrate is −0.243 V, and the corrosion current density is the largest among the tested samples. After laser marking treatment, the corrosion current density decreases due to the generation of an oxide film. The sample corrosion resistance is greatly enhanced when further applying anodizing treatment. An inert TiO_2_ nanotube film is uniformly generated on the laser marked sample, protecting the underlying substrate from corrosion attack. FAS modification is used to modify the samples chemically, and the two modified superhydrophobic surfaces reveal a lower corrosion current density. Due to the awarded water repellency, the modified superhydrophobic surface could generate a lot of air pockets between the solution and the sample surface, thereby significantly reducing the contact area in the corrosion environment and increasing the corrosion resistance.

### 3.3. Superhydrophobic Surface Stability

**Figure 7:**
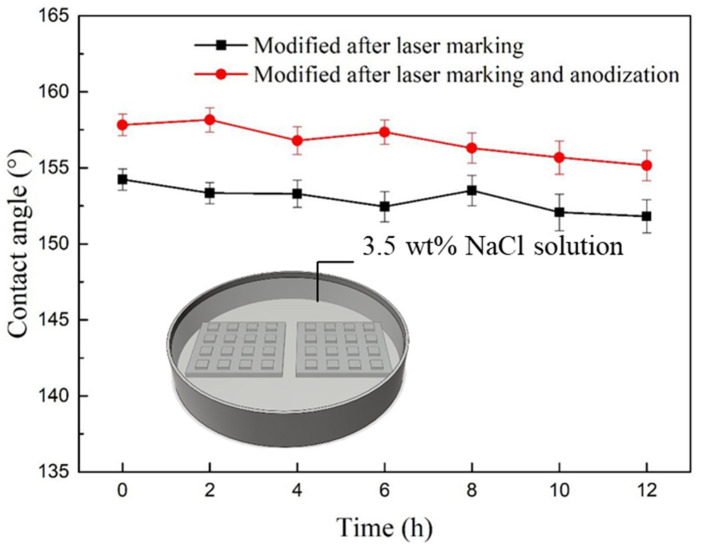
The WCA on the superhydrophobic surfaces after immersing in 3.5 wt% NaCl solution recorded every 2 h.

The superhydrophobic surface needs good stability to suit various fields in practical applications. The chemical stability of surface superhydrophobicity was tested by immersing the sample in 3.5 wt% NaCl solution for 12 h and recording the WCA every 2 h. Figure 7 shows that the WCA of the two superhydrophobic samples hardly decreases after 12 h, which is attributed to the excellent water repellency of the superhydrophobic surface.

**Figure 8:**
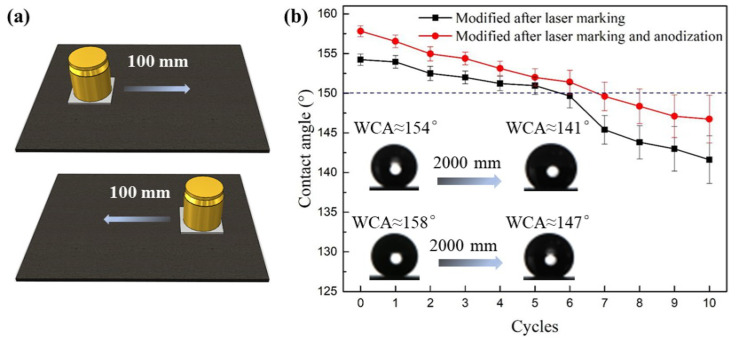
Mechanical stability test of the superhydrophobic surface. (**a**) the testing scheme, (**b**) WCA value after different testing cycles. In each testing cycle, the sample with 100 g weight is uniformly pushed 100 mm forward and pulled 100 mm back to the original position.

This work uses a linear abrasion test method to determine the mechanical stability of superhydrophobic surfaces, as shown in Figure 8. The testing scheme is illustrated in Figure 8a: the sample with 100 g weight load is pushed or pulled on the 600# SiC sandpaper at a constant speed. The superhydrophobic surface and the rough sandpaper are rubbed uniformly, aiming to determine the mechanical stability of sample superhydrophobicity.

Figure 8b presents the recorded WCA values for the superhydrophobic surfaces after different mechanical testing cycles. After five cycles, the laser-marked superhydrophobic surface maintains a contact angle of more than 150°. The WCA decreases to 141° when the testing distance reaches 2000 mm (i.e., 10 cycles), yet the surface still has good hydrophobic properties. The laser marking alters the TC4 surface into a morphology consisting of peaks and valleys. This uneven structure can accommodate the generated wear debris and reduce the abrasive wear, thereby improving the mechanical stability of surface features [27]. In addition, such a microstructure with evenly distributed peaks provides better mechanical performance, which helps maintain the superhydrophobic surface [10].

The anodizing step further improves the mechanical stability of surface superhydrophobicity. The surface maintains superhydrophobicity after 6 testing cycles, and the contact angle of the sample becomes 147° when the testing distance reaches 2000 mm. The enhanced mechanical performance can be attributed to the formation of the inert TiO_2_ nanotube layer on the laser-marked surface. This layer is well-generated and adequately covered on the sample surface. Such a layer can better store debris under wearing tests, and the inert nanotube structure is tough to peel off [37]. Furthermore, the unworn nanotube structure could provide extra air cushion effects for maintaining the surface superhydrophobicity.

## 4. Conclusions

This study manufactured a superhydrophobic surface with enhanced properties on titanium alloys. A combined preparation method of laser marking and anodizing is developed to construct favored surface nanostructures. After the anodizing process, a thin, inert nanotube film grows on the laser-marked surface and provides additional superhydrophobicity, revealing a high WCA at 158°. This anodizing step also gives the sample better corrosion resistance due to its better superhydrophobicity and increased surface uniformity, showing a stable WCA of ~155° throughout the corrosion tests. Besides, the well-designed wear test shows the superior abrasion resistance of the sample after laser marking and anodizing. The inert nanotube film protects the surface features well, and the sample presents good hydrophobicity with a WCA of 147° after the wear tests.

## Figures and Tables

**Table 1:** Tafel polarization parameters of the samples at different processing stages.

Sample	E_corr_ (V_SCE_)	i_corr_ (A/cm^2^)	Corrosion Rate (mm/a)
TC4 substrate	−0.243	1.37 × 10^−^^5^	0.161
After laser marking	−0.289	9.88 × 10^−6^	0.116
After laser marking & anodizing	−0.184	4.72 × 10^−6^	0.055
After laser marking & FAS modification	−0.168	4.34 × 10^−8^	5.096 × 10^−4^
After laser marking, anodizing & FAS modification	−0.01	7.66 × 10^−9^	8.994 × 10^−5^

## References

[1] Zheng S., Yang Y., Tan J., Chen L., Wang Y., He Z. (2022). The hierarchical surface on AZ31 magnesium alloy: Preparation, properties, and performance. Int. J. Mod. Phys. B.

[2] Wang Y., Guan L., He Z., Tan J., Singh H., Hayat M.D., Yao C. (2021). Preparation and characterisation of AAO/Ni/Ni superhydrophobic coatings on aluminium alloys. Surf. Eng..

[3] Dalawai S.P., Saad Aly M.A., Latthe S.S., Xing R., Sutar R.S., Nagappan S., Ha C.-S., Kumar Sadasivuni K., Liu S. (2020). Recent Advances in durability of superhydrophobic self-cleaning technology: A critical review. Prog. Org. Coat..

[4] Geyer F., D’Acunzi M., Sharifi-Aghili A., Saal A., Gao N., Kaltbeitzel A., Sloot T.-F., Berger R., Butt H.-J., Vollmer D. (2020). When and how self-cleaning of superhydrophobic surfaces works. Sci. Adv..

[5] Barthwal S., Lim S.-H. (2021). A durable, fluorine-free, and repairable superhydrophobic aluminum surface with hierarchical micro/nanostructures and its application for continuous oil-water separation. J. Membr. Sci..

[6] Xu Y., Wang G., Zhu L., Shen L., Zhang Z., Ren T., Zeng Z., Chen T., Xue Q. (2021). Multifunctional superhydrophobic adsorbents by mixed-dimensional particles assembly for polymorphic and highly efficient oil-water separation. J. Hazard Mater..

[7] Gao H., Jian Y., Yan Y. (2021). The effects of bio-inspired micro/nano scale structures on anti-icing properties. Soft Matter.

[8] Qi Y., Yang Z., Huang W., Zhang J. (2021). Robust superhydrophobic surface for anti-icing and cooling performance: Application of fluorine-modified TiO2 and fumed SiO2. Appl. Surf. Sci..

[9] Lin Y., Han J., Cai M., Liu W., Luo X., Zhang H., Zhong M. (2018). Durable and robust transparent superhydrophobic glass surfaces fabricated by a femtosecond laser with exceptional water repellency and thermostability. J. Mater. Chem. A.

[10] Wang Y., Guan L., He Z., Zhang S., Singh H., Hayat M.D., Yao C. (2021). Influence of pretreatments on physicochemical properties of Ni-P coatings electrodeposited on aluminum alloy. Mater. Des..

[11] Liu A.-h., Xu J.-l. (2018). Preparation and corrosion resistance of superhydrophobic coatings on AZ31 magnesium alloy. Trans. Nonferrous Met. Soc. China.

[12] Yin X., Yu S., Bi X., Wang L., Zang J., Zhao Y., Wang B. (2021). Preparation of durable, self-cleaning and photocatalytic superhydrophobic Ni3S2 coating on 304 stainless steel surface against contaminations. J. Mater. Sci..

[13] Liu S., Shin Y.C. (2019). Additive manufacturing of Ti6Al4V alloy: A review. Mater. Des..

[14] Lu J., Lu H., Xu X., Yao J., Cai J., Luo K. (2020). High-performance integrated additive manufacturing with laser shock peening -induced microstructural evolution and improvement in mechanical properties of Ti6Al4V alloy components. Int. J. Mach. Tools Manuf..

[15] Abd El-Kader M.F.H., Elabbasy M.T., Adeboye A.A., Zeariya M.G.M., Menazea A.A. (2021). Morphological, structural and antibacterial behavior of eco-friendly of ZnO/TiO2 nanocomposite synthesized via Hibiscus rosa-sinensis extract. J. Mater. Res. Technol..

[16] Jianbing M., Xiaojuan D., Yugang Z., Rufeng X., Xue B., Haian Z. (2019). Fabrication of a Low Adhesive Superhydrophobic Surface on Ti6Al4V Alloys Using TiO₂/Ni Composite Electrodeposition. Micromachines.

[17] Ge He S.L., Wenguo X., Tianlong Y., Jingyan L., Tanlong D. (2018). Durable superhydrophobic Zn ZnO TiO2 surfaces on Ti6V4V substrate with self-cleaning property and switchable wettability. Ceram. Int..

[18] Lu Y., Sathasivam S., Song J., Crick C.R., Parkin I.P. (2015). Repellent materials. Robust self-cleaning surfaces that function when exposed to either air or oil. Science.

[19] Shen Y., Tao J., Tao H., Chen S., Pan L., Wang T. (2015). Nanostructures in superhydrophobic Ti6Al4V hierarchical surfaces control wetting state transitions. Soft Matter.

[20] Liu R., Chi Z., Cao L., Weng Z., Wang L., Li L., Saeed S., Lian Z., Wang Z. (2020). Fabrication of biomimetic superhydrophobic and anti-icing Ti6Al4V alloy surfaces by direct laser interference lithography and hydrothermal treatment. Appl. Surf. Sci..

[21] Gao Y., Sun Y., Guo D. (2014). Facile fabrication of superhydrophobic surfaces with low roughness on Ti–6Al–4V substrates via anodization. Appl. Surf. Sci..

[22] Ren Y., Ye W., Liu A., Zhang L., Dong G., Bo W., Ling X. (2018). Preparation and Properties of Superhydrophobic Titanium Alloy with Hierarchical Structure. Rare Met. Mater. Eng..

[23] Sun J., Chen C., Song J., Liu J., Yang X., Liu J., Liu X., Lu Y. (2019). A universal method to create surface patterns with extreme wettability on metal substrates. J. Colloid Interface Sci..

[24] Wang D., Sun Q., Hokkanen M.J., Zhang C., Lin F.Y., Liu Q., Zhu S.P., Zhou T., Chang Q., He B. (2020). Design of robust superhydrophobic surfaces. Nature.

[25] Aldalbahi A., El-Naggar M.E., Ahmed M.K., Periyasami G., Rahaman M., Menazea A.A. (2020). Core–shell Au@Se nanoparticles embedded in cellulose acetate/polyvinylidene fluoride scaffold for wound healing. J. Mater. Res. Technol..

[26] Wang Y., Zhao X., Ke C., Yu J., Wang R. (2020). Nanosecond laser fabrication of superhydrophobic Ti6Al4V surfaces assisted with different liquids. Colloid Interface Sci. Commun..

[27] Xin G., Wu C., Cao H., Liu W., Li B., Huang Y., Rong Y., Zhang G. (2020). Superhydrophobic TC4 alloy surface fabricated by laser micro-scanning to reduce adhesion and drag resistance. Surf. Coat. Technol..

[28] Pu Z., Zhang D., Jing X., Yang Z., Yang C., Ehmann K.F. (2020). Fabrication of super-hydrophobic and highly oleophobic Ti-6Al-4 V surfaces by a hybrid method. Mater. Res. Bull..

[29] Wang Y., Zhao W., Wu Y., Liu G., Wu X. (2018). Micro/nano-structures transition and electrochemical response of Ti-6Al-4V alloy in simulated seawater. Surf. Topogr. Metrol. Prop..

[30] Plummer D.M., Göke S., Rauber R.M., Di Girolamo L. (2010). Discrimination of Mixed- versus Ice-Phase Clouds Using Dual-Polarization Radar with Application to Detection of Aircraft Icing Regions. J. Appl. Meteorol. Climatol..

[31] Politovich M.K. (1989). Aircraft Icing Caused by Large Supercooled Droplets. J. Appl. Meteorol..

[32] Xiang G.-X., Li S.-Y., Song H., Nan Y.-G. (2020). Fabrication of modifier-free superhydrophobic surfaces with anti-icing and self-cleaning properties on Ti substrate by anodization method. Microelectron. Eng..

[33] Shen Y., Tao H., Chen S., Xie Y., Zhou T., Wang T., Tao J. (2014). Water repellency of hierarchical superhydrophobic Ti6Al4V surfaces improved by secondary nanostructures. Appl. Surf. Sci..

[34] Cassie A., Baxter S. (1944). Wettability of porous surfaces. Trans. Faraday Soc..

[35] Wu Y., Lian J., Wang Y., Sun J., He Z., Gu Z. (2021). Potentiostatic electrodeposition of self-supported Ni S electrocatalyst supported on Ni foam for efficient hydrogen evolution. Mater. Des..

[36] Wu Y., Zhang Y., Wang Y., He Z., Gu Z., You S. (2021). Potentiostatic electrodeposited of Ni–Fe–Sn on Ni foam served as an excellent electrocatalyst for hydrogen evolution reaction. Int. J. Hydrogen Energy.

[37] Wu H., Xie L., Zhang R., Tian Y., Liu S., He M., Huang C., Tian W. (2019). A novel method to fabricate organic-free superhydrophobic surface on titanium substrates by removal of surface hydroxyl groups. Appl. Surf. Sci..

